# Chronic subcutaneous infection of Purpureocillium lilacinum in an immunocompromised patient: Case report and review of the literature

**DOI:** 10.1016/j.mmcr.2022.08.001

**Published:** 2022-09-03

**Authors:** Robin Albert, Adrien Lemaignen, Guillaume Desoubeaux, Eric Bailly, Louis Bernard, Marion Lacasse

**Affiliations:** a Infectious Diseases Unit, Tours, 37000, France; b Mycology Unit, Tours, 37000, France

**Keywords:** Purpureocillium lilacinum, Immunocompromised, Kidney transplantation, Skin infection

## Abstract

Our case reports a 52-year-old woman who presented with *Purpureocillium lilacinum* skin infection after a renal transplantation. The diagnosis was difficult and this species exhibits many resistances to antifungal agents. The clinical history was marked by a relapse causes by a foreign body. Our case suggests that posaconazole may be an alternative to cure *P. lilacinum* infection, and that the surgical debridement, the identification and removal of a foreign body may improve the prognosis.

## 1 Introduction

*Purpureocillium lilacinum,* formerly known as *Paecilomyces lilacinus* [1] is an emerging pathogenic fungus rarely described as a cause of skin infection. Only a few case-reports [2,3] or literature reviews [4] reported this agent as responsible for cutaneous, but also ocular or systemic diseases especially in solid organ or bone marrow transplanted patients, and those under corticosteroid therapy. This infection has been described mainly in immunocompromised patients, but some cases suggest possible infection in immunocompetent patients [5]. *P. lilacinum* is a hyaline hyphomycete with a ubiquitous distribution in the environment, so multiple modes of contamination are possible [1]. Therapeutic difficulties related to the resistance to antifungals by this pathogen are described [6] and the use of new approaches is essential to improve outcomes. This case shows the importance of combined treatment: oral azole medication associated with foreign body search and a complete surgical debridement in a patient with recurrent *P. lilacinum* infection.

## 2 Case presentation

A 52-year-old Caucasian woman received a hepato-renal allograft transplant in December 2015 because of an end-stage autosomal dominant polycystic kidney. She was immunocompromised with mycophenolic acid (1000mg per day), tacrolimus (15mg per day) and prednisone (8mg per day). She didn't present any other predispositions: no malignancies, no hepatic cirrhosis or diabetes mellitus.

**Fig. 1:**
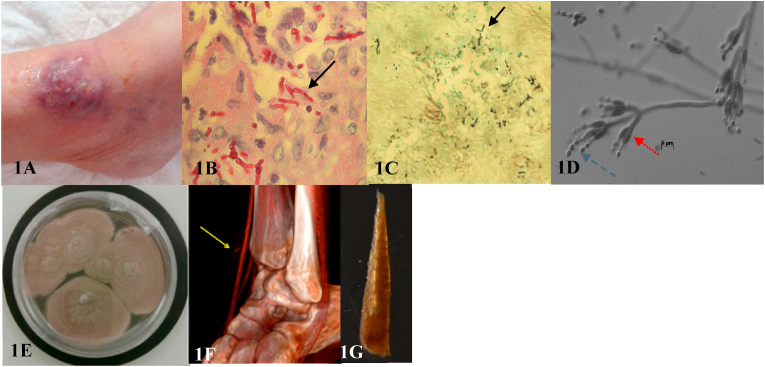
(1A) Macroscopic view of *P. lilacinum's* skin lesion, (1B) Microscopic observation of hyphal elements (black arrow) in the biopsy (periodic acid schiff x40), (1C**)** Microscopic observation of hyphal elements (black arrow) in the skin biopsy (methenamine Gomori silver staining x40), (1D) Microscopic observation of phialides (red arrow) and oblong conidia produced according to an enteroblastic centrifuge mode (blue arrow) (lactophenol cotton blue x200), (1E) Macroscopic observation of *P. lilacinum* colonies on Sabouraud dextro agar plate, (1F) Tomography-scan reconstruction with the view of splinter, (1G) Macroscopic view of splinter after surgery. (For interpretation of the references to colour in this figure legend, the reader is referred to the Web version of this article.)

Four months after the transplantation (day 0), the patient presented with a spontaneous tender skin tumefaction of the internal malleolus of her left ankle. On the 30th day, the lesion became purplish with multiple fistula orifices which excreted a hematic and purulent liquid (Fig. 1). She didn't present any fever, chills, night sweats, general signs or lymphadenopathy and onychomycosis. She was a cashier on disability since her transplant and she lived in a rural area in France without pets. She denied bathing, recent trip abroad, trauma or insect bites before the appearance of the lesion but she reported gardening. The blood count analysis found lymphopenia, but neither neutropenia nor signs of inflammation. Ultrasound examination reported a cystic structure measuring 17 x 6 × 17 mm. Therefore, a punch biopsy was perfomed (day 133).

Histopathology observation showed an intense granulomatous dermatitis with necrosis and many filaments compatible with a fungal infection (Fig. 1). Bacterial and mycobacterial cultures were negative. Direct and fluorescent microscopic examination with Calcoluor (a fluorescent brightener) was negative, but fungal culture (incubated at 30 and 35°c for 30 days) on Sabouraud dextro agar highlighted several colonies growing in three days (Fig. 1). *Purpureocillium lilacinum* was finally identified (day 139) by macroscopic and microscopic phenotypic characteristics and confirmed by sequencing (gene bank accession number: MF444831, amplification of the D1-D2 regions of 28s RNA and B-tubulin according to the method of O'Donnel, In the fungal holomarph 1993 and Glass et al., Applied and environmental microbiology 1995). Antifungal susceptibility testing showed reduced susceptibility to commonly used antifungal drugs except for posaconazole and voriconazole, with minimal inhibitory concentration (MIC) at 0.25 mg/L for posaconazole and 0,5 mg/L for voriconazole (Table 1).

**Table 1:** MIC in liquid medium according to EUCAST from National Reference Centre Pasteur Institute.

Molecule	MIC
Amphotericin B	≥4 mg/L
Itraconazole	≥8 mg/L
Voriconazole	0,5 mg/L
Posaconazole	0,25 mg/L
Isavuconazole	2 mg/L
Caspofungin	≥4 mg/L
Micafungin	≥4 mg/L
Flucytosin	Not done
Fluconazole	Not done

The thoraco-abdominal-pelvian scan and fundus examination did not find secondary localizations, neither deep-seated infection nor bone invasion (day 173). Fungal blood cultures remained sterile (after 14 days of incubation) and BDglucan serology was negative. Only a small elongated foreign body was noticed near the *flexor retinaculum* on the tomography-scan reconstruction images, which was compatible with a splinter (Fig. 1). All investigations have been done prior to the initiation of treatment.

A medical treatment without surgery was initiated five months after lesion discovery (day 166) for at least six months with 300mg posaconazole *per* day. At the end of the treatement, clinical evolution was favourable with the resolving of the cutaneous lesion. Nonetheless, one week after the patient relapsed (day 356).

Two months after the relapse (day 417), a second lign of treatement was introduced with 300mg posaconazole *per* day during three months combined with complete extraction of the foreign body during surgical intervention (Fig. 1). A second local relapse of the lesion was observed two weeks after the end of the medication (day 522). The treatement with posaconazole was extended for six additional months. We performed therapeutic monitoring by regular measurement of blood levels of posaconazole and tacrolimus. A strong interraction between azoles and anticalcineurins was noticed, hence we have been forced to reduce the dosage of tacrolimus by 80%. Our patient presented adverse effects due to posaconazole: digestive disorders and phototoxicity. The patient has had a three years remission since then.

## 3 Discussion

*Purpureocillium lilacinum*, a fast-growing environmental mold, has been described as an emerging pathogen in immunocompromised patients [7]. Nowadays, its medical management is not consensual. In the present case*,* we discuss the diagnostic and therapeutic tools and the importance of searching for fungus vehicle in order to enhance the curative strategy.

*Purpureocillium lilacinum* is a hyaline hyphomycete that exists worldwide especially in soil, decaying food and paper. It is considered as a rare cause of infection (101 cases in the literature [8]) in both immunocompromised and immunocompetent patient [5]. The most common risk factors identified are haematological and oncological diseases, steroid treatment, solid organ transplantation and diabetes mellitus [8]. Reviews suggested that *P. lilacinum* has a moderate virulence, but that several predispositions such as immunosuppression (because of depletion of macrophages, neutrophils, interleukin and nitric oxide synthase in the tissue [9]), implants, or foreign materials [4] can heighten the virulence and dissemination of the agent. One article highlighted that this species could be able to produce a biofilm [1], which strengthens our arguments exposed in our case to research a skin inoculation of a biological foreign body especially in chronic infection or relapse. Many cases demonstrated a direct skin inoculation: by a tattoo [3], contaminated skin lotion on a skin abrasion [9], catheters or a splinter in our subject.

Main sites of infections described in the literature in descending order are eye structures (oculomycosis, keratitis and endophthalmitis), then skin, and finally lung, soft tissue, bloodstream, and sinuses [8]. As mentioned before, common gateways are breach of the skin barrier by inoculation, or inhalation [7]. Clinical cutaneous and sub-cutaneous presentations are varied: single or grouped, usually pseudo-verrucous lesions, erythematous macules, papules, or vesicles, soft or indurated nodules with or without necrosis, cellulitis or ulcers [7]. The cutaneous location is usually distributed on the limb’ extremities [2] which reinforces the inoculation hypothesis. Compared to systemic infection, the cutaneous damage is not associated with fever or other general features. That's why extension work up is not consensual, and a local evaluation by echography, MRI or CT-scan may be sufficient. Due to a local invasion without systemic spread, the mortality of the skin infection is lower than in the other sites.

The microbiological diagnosis is confirmed by histological or direct examination of a biopsy associated with positive culture [5]. As observed in our case, histological examination shows classically a granuloma and filamentous fungus with septate hyphae [10] respectively with PAS and Gocrott-Gomori staining. The culture on Sabouraud-dextrose-agar produces woolly colonies with a slight pink tint that grew rapidly at room temperature [4]. The microscopic observation following with Lactophenol cotton blue (LPCB) staining shows characteristic reproductive structures [10]: the budding of a mother cell gives rise to short septate hyphae which carry phialides, and the phialides produce unicellular oblong conidia. This cellular mechanism explains the ability of this agent to sporulate in infected tissues. Regarding the complexity of identifying fungal agents on morphological structures with microscopy, it is recommended [5] to confirm the species identification by DNA sequencing, or specific Polymerase Chain Reaction (PCR) amplification of the 18S ribosomal ribonucleic acid (18S RNA) [3], this is particularly justified because there are differences in sensitivity to antifungals within the same species, for example between *Purpureocillium variotii* and *P. lilacinum* [6].

This fungal agent is known to have inherent low antifungal susceptibilities [8,11]: amphotericin B, fluconazole and flucytosine. Itraconazole [8] is less active *in vitro* than voriconazole or posaconazole but it is predominantly administrated, with some clinical success. Echinocandins show variable MICs. Based on the literature at the date of our case, treatment is not consensual, but some cases reported clinical success of voriconazole [6,12,13] and in two sporadic cases with posaconazole [13,14], including one after failure with voriconazole. A study [15] suggested that association of voriconazole and terbinafine has a synergistic interaction in *in vitro* culture, but it is still of debate. Another case presented a new perspective of treatment with the success of isavuconazole [13] which can be used in patient's intolerant to voriconazole or posaconazole. The American Food and Drug Agency (FDA) suggests that isavuconazole and posaconazole are better tolerated than voriconazole with less neurological disorders (hallucinations, peripheral neuropathies), skin toxicity and interaction with immunosuppressive treatment [7,16]. We chose posaconazole in our case, and that the *in vitro* antifungal susceptibility is higher with posaconazole, and the tolerance is better than with voriconazole. In our subject, the tolerance of posaconazole was relative (phototoxicity and digestive disorders).

This case also demonstrated that medication alone is insufficient in the event of a relapse and should be combined with surgery for the debridement of the infected tissue. Other studies demonstrated that surgery alone or associated with medication [2] is an efficient treatment to prevent relapse and shorten the duration of treatment. The originality of this case lies in the discovery of a foreign body under the skin and that its persistence was the cause of a clinical relapse. Therefore, it seems very useful to carefully search the vehicle and a foreign body to prevent recurrence. The duration of the treatment is not clear, it can depend on adequate surgical resection, inoculum size and drug sensibilities, but in the literature [3,13] and in our case, a prolonged treatment of at least three months is often noted.

Also, this article describes, as with another case [16], the necessity of the reduction of immunosuppressive drugs posology and pharmacological assay for the therapeutic adjustment. In fact, azoles like posaconazole are enzymatic inhibitors of the cytochrome CYP3A4, which particularly interacts with anticalcineurins.

In conclusion, we suggest that a combined treatment with the new azoles like posaconazole, complete surgical intervention, and foreign body search and removal could have a beneficial effect on recurrences or relapses of cutaneous infections caused by *P. lilacinum*.

## Ethical statement for solid state ionics

Hereby, I Robin ALBERT consciously assure that for the manuscript “Chronic subcutaneous infection of Purpureocillium lilacinum in an immunocompromised patient: case report and review of the literature” the following is fulfilled:

1) This material is the authors' own original work, which has not been previously published elsewhere.
2) The paper is not currently being considered for publication elsewhere.
3) The paper reflects the authors' own research and analysis in a truthful and complete manner.
4) The paper properly credits the meaningful contributions of co-authors and co-researchers.
5) The results are appropriately placed in the context of prior and existing research.
6) All sources used are properly disclosed (correct citation). Literally copying of text must be indicated as such by using quotation marks and giving proper reference.
7) All authors have been personally and actively involved in substantial work leading to the paper, and will take public responsibility for its content.

The violation of the Ethical Statement rules may result in severe consequences.

To verify originality, your article may be checked by the originality detection software iThenticate. See also http://www.elsevier.com/editors/plagdetect.

I agree with the above statements and declare that this submission follows the policies of Solid State Ionics as outlined in the Guide for Authors and in the Ethical Statement.

## Declaration of competing interest

The authors declare that there is no conflict of interest regarding the publication of this article.

All authors contributed equally.
